# Malaria epidemics associated with low net use in preadolescent and young adult population in Dielmo (Senegal), a malaria pre-elimination area

**DOI:** 10.1186/s13071-024-06172-1

**Published:** 2024-02-19

**Authors:** Amélé Nyedzie Wotodjo, Souleymane Doucoure, Nafissatou Diagne, Fatoumata Diene Sarr, Cheikh Sokhna

**Affiliations:** 1 UMR VITROME (Vecteurs - Infections Tropicales et Méditerranéennes) Campus International IRD-UCAD, Dakar, Senegal; 2 Institut Pasteur de Dakar, 36 Avenue Pasteur, 220 Dakar, Senegal; 3 Aix-Marseille Univ, IRD, AP-HM, SSA, VITROME, Marseille, France

**Keywords:** Malaria upsurge, Net ownership, Net use, Older children, Young adult, Dielmo, Senegal

## Abstract

**Background:**

The epidemic rebounds observed in 2010 and 2013 in Dielmo, a Senegalese village, during a decade (2008–2019) of universal coverage using a long-lasting insecticidal net (LLIN) strategy could have contributed to the resurgence of malaria. Thus, this study was undertaken to understand the implications of net ownership and use on malaria rebound events.

**Methods:**

A longitudinal study was carried out in Dielmo with 11 years of LLIN implementation from July 2008 to June 2019 with successive net renewals in 2011, 2014, 2016 and 2019. Quarterly cross-sectional surveys were performed to assess LLIN ownership and use by different age groups in the population. In addition, malaria incidence and transmission were assessed during the study period.

**Results:**

Ownership of LLINs decreased significantly from 88% in the 1st year of net implementation to 70% during the first malaria upsurge and 72% during the second upsurge while net use decreased significantly from 66% during the 1st year to 58% during the first malaria upsurge and 53% during the second upsurge.

Among young adults aged 15–29 years, net use decreased significantly from the 2nd year (51%) of net use to reach 43% during the first malaria upsurge and only 32% use during the second malaria upsurge. During the second malaria upsurge, net use was significantly lower among older children aged 10–14 years old than during the 1st year of net use (*p* < 0.001). During the first and the second malaria upsurges, the malaria incidence was significantly higher among children aged 10–14 years old (0.4 attacks per person-year) and young adults aged 15–29 years old (0.3 and 0.4 attacks per person, respectively) than during that the 1st year of net implementation (only 0.02 attacks per person-year for 10–14 year olds and 0.04 for 15–29 year olds; *p* < 0.001).

**Conclusions:**

The first malaria upsurge occurred following a progressive decrease in net use after the 2nd year of their implementation with an important increase in malaria incidence among older children while the second malaria upsurge was significantly associated with the decrease of net use among older children and young adults. The regular use of nets in all age groups prevented the occurrence of a third malaria upsurge in Dielmo.

**Graphical Abstract:**

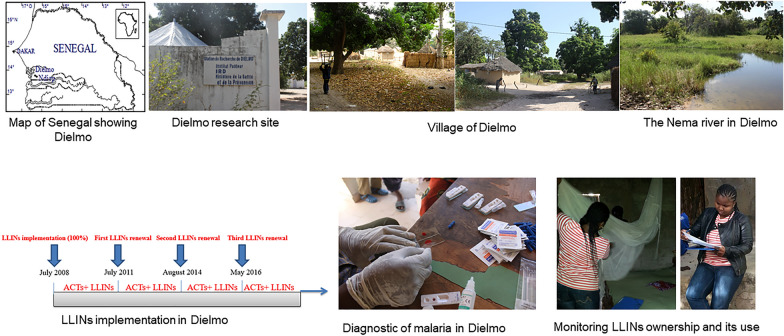

## Background

The widespread use of long-lasting insecticidal nets (LLINs) in malaria-endemic countries has led to a decrease of the disease. The positive effect of net use in decreasing the malaria burden could be undermined by vector resistance to insecticides used for bed net impregnation [1]. The loss of durability occurring over time with a reduction in the amount of insecticide present and physical degradation of the net could also impair LLINs' efficacy [2, 3]. The ownership and use of mosquito nets are critical parameters for the success of universal LLIN coverage to decrease malaria transmission [4, 5]. To this end, in addition to vulnerable groups such as pregnant women and children < 5 years old, current strategies target the entire population in malaria-endemic areas. In Dielmo, a Senegalese malaria-endemic village, universal coverage of LLINs has been implemented since July 2008 with successive renewal until 2019 to such an extent that the level of pre-elimination has been reached. However, although a sharp reduction of malaria cases was observed, two malaria upsurges occurred during the period of net implementation in this village [6, 7]. However, LLIN use was still protective against the occurrence of malaria during these malaria upsurges [8]. Although sustainable universal coverage with LLINs can protect users in case of malaria outbreaks, it is essential to clearly characterize the population groups in which the disease occurs to identify gaps in the collective protection provided by the nets. The aim of this study was to assess net ownership and use in the Dielmo population by age group and its implications for the occurrence of malaria resurgences.

## Methods

### Dielmo village and the study procedure

The Dielmo research site has been described in detail elsewhere [9]. Briefly, the village is located in a Sudan savannah region of central Senegal, 280 km southeast of Dakar. Malaria transmission is currently seasonal from July to October each year, and the disease is at the pre-elimination stage [8]. In July 2008, the universal net coverage strategy was implemented for the first time in Dielmo, and LLINs were offered to all residents. In July 2011, August 2014, May 2016 and June 2019, the universal coverage was repeated by completely renewing all LLINs. Before renewal, the old LLINs from the previous campaign were taken away to ensure that there was no concomitant use of nets from different universal coverage campaigns. During the distribution campaigns in 2008, 2011 and 2014, only Permanet^®^ 2.0 (active ingredient: deltamethrin) LLINs were given to the residents. In the 2016 campaign, Permanet^®^ 2.0 with Olyset^®^ (active ingredient: permethrin) LLINs were distributed in Dielmo; in 2019, only Yorkol^®^ LLINs were used (active ingredient: deltamethrin). During each distribution campaign, one net was allocated to each sleeping place. A sleeping place was defined as the place where individuals in the household were sleeping. It could be occupied by a single individual or several. Simultaneously to the introduction of LLINs, each participating household was visited quarterly in the morning by project technicians to assess the bed net use. Individuals were asked whether they had used nets the night preceding the visit and whether they never, always or sometimes used nets. Two groups were created for net use: those who always used nets and those who did not consistently use nets. This second category grouped individuals who reported “sometimes” or “never” using LLINs during the corresponding quarter as well as individuals who did not have LLINs. This evaluation covered 11 years of follow-up of net use from the time the nets were first implemented in July 2008 to the last universal coverage campaign in June 2019. Each year of net use was defined from July of a year to June of the next year, as shown in Table 1. During this period, human landing catch (HLC) method was performed monthly to assess malaria transmission. The artesunate plus amodiaquine combination was used to treat clinical malaria attacks during the study period. Thick smears stained with Giemsa and rapid diagnosis test (RDT) and polymerase chain reaction test were performed to determine the presence of the malaria parasite for the diagnostics. The clinical malaria attack incidence rate was calculated as the ratio of the number of clinical malaria attacks recorded divided by the number of person-days of follow-up during a given period. Analyses were performed using Stata software, version 11.0 (College Station, TX, USA). The chi-square test for incidence rates was used to compare the incidence rate for each year. To consider the interdependence of successive observations in the same individual, random logistic regression was used to assess the effect of sex, age group, rainfall (defined as the cumulative number of mm of rainfall during the quarter) and malaria transmission on using nets in the Dielmo population. Age group was classified into six groups: < 5; 5–9; 10–14; 15–29; 30–44; ≥ 45 years, according to the literature [6, 9].

**Table 1:** Distribution of the years of net implementation in Dielmo

July 8–June 9	July 9–June 10	July 10–June 11	July 11–June 12	July 12–June 13	July 13–June 14	July 14–June 15	July 15–June 16	July 16–June 17	July 17–June 18	July 18–June 19
Year 1	Year 2	Year 3	Year 4	Year 5	Year 6	Year 7	Year 8	Year 9	Year 10	Year 11
1st year of net implementation	2nd year of net implementation	First malaria upsurge	First net renewal		Second malaria upsurge	Second net renewal	Third net renewal in May 2016			

### Ethical considerations

Written informed consent was obtained from all participants or the guardians of the minors/children enrolled in the study. The study was approved by the Ministry of Health of Senegal, the assembled village population and the National Ethics Committee of Senegal.

## Results

### Universal LLIN coverage and malaria morbidity and transmission

**Fig. 1:**
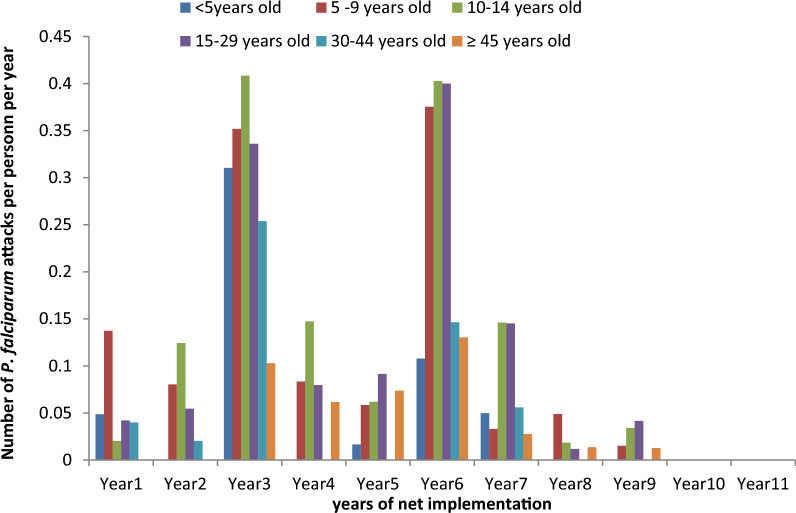
Malaria incidence by year of net implementation and age group in Dielmo

During the first and 2nd years of net implementation in this village, the incidence of malaria was 0.05 and 0.04 attacks per person per year, respectively. An upsurge of malaria was observed during the 3rd year of net use, and the incidence of malaria increased to 0.30 attacks per person per year (*p* < 0.001). The malaria incidence was the highest among the children aged 10–14 years old with 0.4 attacks per person-year (Fig. 1), which was significantly higher than that observed during the 1st year of net implementation (only 0.02 attacks per person-year; *p* < 0.001). All LLINs were renewed for the first time in July 2011. After that, malaria consequently decreased during the 1st and 2nd year after the nets had been renewed (mean 0.05 attacks per person per year during these 2 years; *p* < 0.001). Again, an upsurge occurred the 3rd year after nets were renewed, and the incidence of malaria increased to 0.26 attacks per person per year (*p* < 0.001). During this second malaria upsurge, the malaria incidence was higher in the 10–14-year-old and 15–29-year old age groups, with 0.4 attacks per person-year, than that observed during the year preceding the occurrence of the second upsurge (0.06 and 0.09 attacks per person-year among 10–14 year olds and 15–29 year olds, respectively).

All nets were renewed for a second time in August 2014, and malaria incidence decreased from 0.26 to 0.08 and 0.01 attacks per person per year in the first and 2nd year following the second net renewal (*p* < 0.001). In response to the two previous malarial upsurges, the universal LLIN coverage was renewed for the third time in May 2016, < 2 years after the second renewal and before the beginning of the rainy season. The incidence of malaria was only 0.02 attacks per person per year in the 1st year following the third net renewal. During the 2nd and 3rd year following the third net renewal, which corresponded to 10 and 11 years, respectively, since nets were first introduced into the village of Dielmo, no clinical malaria attack was observed till the nets were renewed for the fourth time in June 2019 in the whole country following the directives of the National Malaria Control Program of Senegal (Fig. 1).

**Fig. 2:**
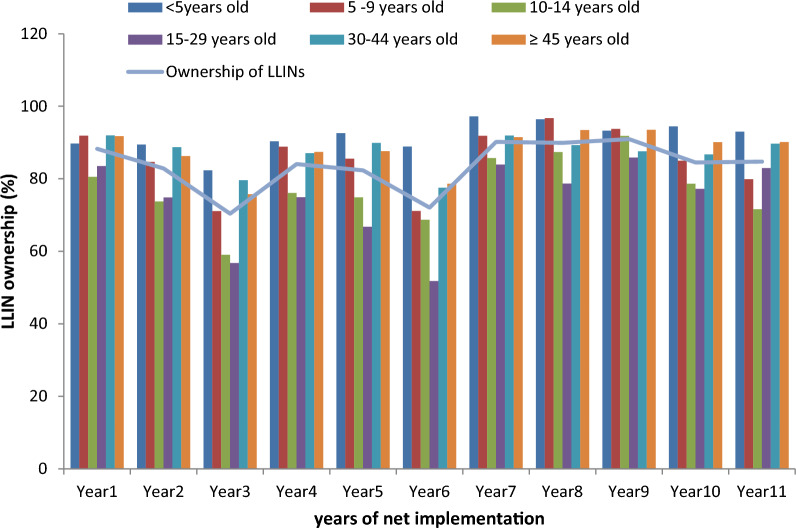
Net use by age group; global net use; rainfall and malaria transmission by year of net implementation in Dielmo

The entomological inoculation rate (EIR) increased from 35.8 infective bites per person per year during the 1st year of net implementation to 50.0 the 2nd year and 77.5 infective bites per person per year the 3rd year of net implementation (the 3rd year corresponded to the first malaria upsurge period). It decreased to 67.5 and 15.0 infective bites per person per year the 1st and 2nd year after the first net renewal, respectively, and then increased to 40.1 infective bites per person per year during the 3rd year of the first net renewal, which corresponded to the second malaria upsurge period. The EIR again decreased to 15.9 the 1st year of the second net renewal and 0 infective bites per person per year the 2nd year of the second net renewal. The EIR was only 2.5, 1.7 and 0 infective bites per person per year the 1st, 2nd and 3rd year after the third net renewal, respectively (Fig. 2).

### Global net ownership and use

**Fig. 3:**
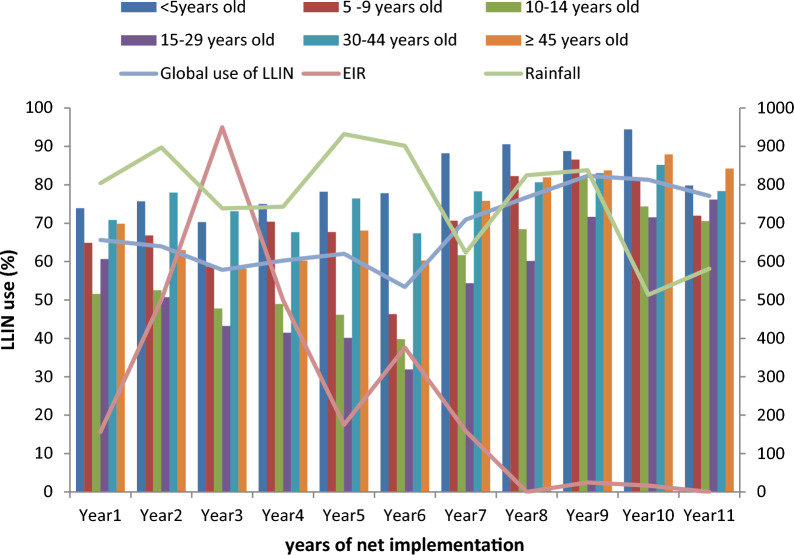
Ownership of LLINs in Dielmo for 11 years by age group

Net ownership was 88% the 1st year of net implementation and 83% the 2nd year. It was 70% during the first malaria upsurge and 72% during the second one. During the 3rd year following the third net renewal, net ownership was still high at 85%. Net use was 66% during the 1st year of net implementation and 64% during the 2nd year. It was only 58% during the first malaria upsurge and 53% during the second one. During the 3rd year following the third net renewal, net use was still high for the first time with 77% (Figs. 2 and 3).

When controlling factors such as rainfall, age and sex, the level of global net use during the 1st year of net implementation did not significantly differ from that during the 2nd year (*p* = 0.63) (Table 2). However, net use decreased significantly from the 3rd year of net implementation (during the first malaria upsurge) (58%) to reach its lowest level during the 6th year of net use (during the second malaria upsurge) (53%). Indeed, from the 3rd to 6th year of net implementation, net use was significantly lower than during the 1st year of net use (*p* < 0.001 during the 3rd, 4th and 6th years, *p* = 0.005 during the 5th year). During the 7th year of net use, which corresponded to the year when nets were renewed for the second time, net use was not significantly different from that during the 1st year of net implementation (*p* = 0.23). From the 8th to 11th years, net use, which was at least 77%, was significantly higher than in the 1st year of net implementation (*p* < 0.001). Net use was significantly higher on rainy days (*p* < 0.001) and among women (*p* < 0.001) (Table 2). Overall, women used nets almost twice as often as men (Table 2).

**Table 2:** Random effect logistic regression models exploring factors associated with LLIN use in Dielmo

Characteristics	Subcategory	Number of observations *n* = 6.558 *n* (%)	Use of LLINs		Univariate analysis		Multivariate analysis	
			No *n* = 4.233 *n* (%)	Yes *n* = 9.301 *n* (%)	IRR (95% CI)	*P*-value	aIRR (95% CI)	*P*-value
Year of LLIN use								
	1st year of LLIN use (ref)	1.373 (9.92)	432 (10.21)	901 (9.69)	1		1	
	2nd year of LLIN use	1.451 (10.48)	458 (10.82)	928 (9.98)	0.93 (0.77–1.13)	0.48	0.95 (0.78–1.16)	0.63
	3rd year of LLIN use	1.461 (10.56)	556 (13.13)	845 (9.09)	0.58 (0.47–0.70)	< 0.001	0.65 (0.53–0.79)	< 0.001
	Fourth year of LLIN use	1.411 (10.2)	530 (12.52)	850 (9.14)	0.60 (0.50–0.74)	< 0.001	0.68 (0.56–0.84)	< 0.001
	Fifth year of LLIN use	1.465 (10.59)	522 (12.33)	909 (9.77)	0.67 (0.55–0.82)	< 0.001	0.75 (0.61–0.92)	0.005
	Sixth year of LLIN use	1.306 (9.44)	587 (13.87)	697 (7.49)	0.34 (0.28–0.42)	< 0.001	0.36 (0.29–0.45)	< 0.001
	Seventh year of LLIN use	1.118 (8.08)	310 (7.32)	793 (8.53)	0.94 (0.76–1.17)	0.61	1.15 (0.92–1.44)	0.23
	Eight year of LLIN use	1.358 (9.81)	303 (7.16)	1.042 (11.2)	1.33 (1.08–1.65)	0.008	1.95 (1.56–2.43)	< 0.001
	Ninth year of LLIN use	1.279 (9.24)	213 (5.03)	1.053 (11.32)	1.94 (1.55–2.43)	< 0.001	2.55 (2.01–3.23)	< 0.001
	Tenth year of LLIN use	845 (6.11)	150 (3.54)	687 (7.39)	1.93 (1.49–2.49)	< 0.001	2.96 (2.26–3.89)	< 0.001
	Eleventh year of LLIN use	773 (5.59)	172 (4.06)	596 (6.41)	1.30 (1.01–1.68)	0.041	2.33 (1.79–3.04)	< 0.001
Socio-demographic characteristics								
Age group								
	< 5 years (ref)	2.268 (16.39)	400 (9.45)	1.810 (19.46)	1		1	
	5–10 years	2.316 (16.73)	661 (15.62)	1.601 (17.21)	0.60 (0.49–0.72)	< 0.001	0.45 (0.37–0.56)	< 0.001
	10–14 years	1.869 (13.5)	755 (17.84)	1.067 (11.47)	0.43 (0.34–0.55)	< 0.001	0.27 (0.21–0.34)	< 0.001
	15–29 years	2.870 (20.74)	1,292 (30.52)	1.522 (16.36)	0.40 (0.31–0.51)	< 0.001	0.20 (0.15–0.26)	< 0.001
	30–44 years	1.940 (14.02)	441 (10.42)	1.469 (15.79)	0.76 (0.55–1.04)	0.09	0.40 (0.28–0.57)	< 0.001
	≥ 45 years	2.577 (18.62)	684 (16.16)	1.832 (19.70)	1.02 (0.71–1.46)	0.93	0.44 (0.32–0.69)	< 0.001
Sex								
	Male (ref)	6.783 (49.01)	2.267 (53.56)	4.362 (46.9)	1		1	
	Female	7.057 (50.99)	1.966 (46.9)	4.939 (53.1)	1.64 (1.23–2.20)	0.001	1.89 (1.42–2.51)	< 0.001
Rainfall								
					1.0014 (1.0011–1.0015)	< 0.001	1.0015 (1.0014–1.0017)	< 0.001
Entomogical inoculation rate (EIR)					1.0018 (0.998–1.006)	0.35		

### Net ownership and use by age

During the first malaria upsurge, net ownership was 82, 59 and 57% among children < 5 years old, those 10–14 years old and those 15–29 years old, respectively (Fig. 3). For each of these age groups, the level of net use was 70, 48 and 43%, respectively (Fig. 2). In the 30–44-year-old and ≥ 45-year-old age groups, net ownership was 80% and 76% with a 73% and 58% level of use, respectively.

During the second malaria upsurge, net ownership was 89% among children < 5 years old and 71% among children aged 5–9 years old while it was only 69% among children aged 10–14 years old and 52% among young adults of 15–29 years old. The level of net use was 78% among children < 5 years old, 46% among those aged 5–9 years old, 40% among children aged 10–14 years old and 32% among young adults of 15–29 years old.

Three years after the third universal coverage campaign, a high level of net ownership (85%) and use (77%) was observed in almost all age groups. Among those < 5 years old, net ownership was 93% while net use was 80%. Even among 10–14 year olds, net ownership and use were 72% and 71% while among 15–29 year olds net ownership was 83% and net use 76%. Among the remaining age groups, net ownership was at least 80% and net use at least 72%. Since the third renewal and onwards, net use was > 70% regardless of the age group.

## Discussion

The sustainable implementation of universal LLIN coverage in Dielmo has led to an important decrease in the malaria transmission level marked by reduced asymptomatic parasite carriage within the community [8, 10]. This important decrease in the disease has undoubtedly resulted in reaching the pre-elimination level up to the elimination of the disease as no local malaria case was observed between 2018 and 2021. Malaria upsurges seem to be associated with a decrease in global net use especially in older children and young adults. During malaria upsurges, older children (10–14 years old) and young adults (15–29 years old) were more vulnerable than the rest of the population as their incidence of malaria increased significantly compared with that observed during the year before net implementation in this village [11]. During the two malaria upsurges, the malaria patients used their nets significantly less than non-malaria patients, and approximately half of malaria patients were older children and young adults [4, 8]. The non-regular use of nets among this sub-category of the population could be due to their behavior as they tended to stay outside late at night to watch television or to stroll around compared to the rest of Dielmo's population [8]. Some studies previously showed that this outdoor behavior is a malaria risk factor [12, 13]. In Dielmo, following the implementation of LLINs, the residual vector populations of *Anopheles gambiae* sensu lato and *An. funestus* had an increased preference for biting outdoors [14]; thus, spending time out late at night could increase the risk of contracting malaria in this village [7]. This observation among older children and young adults needs to be considered when defining new strategies for malaria elimination, especially in areas where malaria has decreased significantly, to avoid malaria resurgences.

Following the third net renewal, where an awareness campaign on the use of nets was carried out, no malaria upsurge was observed; moreover, use of nets was > 70% in all age groups during the 3rd year after nets had been renewed. This observation could be explained by the appropriation of the strategy by the villagers over time, shown by their purchasing of nets themselves if needed and by the advice from the older villagers about the benefits of net use. In addition, long-term net use has negatively impacted the abundance of the *Anopheles* population, which has decreased substantially.

In Dielmo, nets have remained effective in preventing malaria during the 3 years of their use [4, 7], and their regularly use in all age groups has prevented the occurrence of further malaria upsurges. Older children and young adults need more attention and advice during universal net coverage campaigns for the success of this tool against malaria.

## Conclusions

The first malaria upsurge occurred following a progressive decrease in net use from the 2nd year of their implementation with an important increase of malaria incidence among older children while the second malaria upsurge was significantly associated with the decrease of net use among older children and young adults. The regular use of nets in all age groups prevented the occurrence of a third malaria upsurge in Dielmo.

## Data Availability

The datasets used and/or analysed during the current study are available from the corresponding author on reasonable request.
